# Generalized Scaling and the Master Variable for Brownian Magnetic Nanoparticle Dynamics

**DOI:** 10.1371/journal.pone.0150856

**Published:** 2016-03-09

**Authors:** Daniel B. Reeves, Yipeng Shi, John B. Weaver

**Affiliations:** 1 Department of Physics and Astronomy, Dartmouth College, Hanover, NH, 03755 United States of America; 2 Department of Radiology, Geisel School of Medicine, Hanover, NH, 03755 United States of America; VIT University, INDIA

## Abstract

Understanding the dynamics of magnetic particles can help to advance several biomedical nanotechnologies. Previously, scaling relationships have been used in magnetic spectroscopy of nanoparticle Brownian motion (MSB) to measure biologically relevant properties (e.g., temperature, viscosity, bound state) surrounding nanoparticles *in vivo*. Those scaling relationships can be generalized with the introduction of a master variable found from non-dimensionalizing the dynamical Langevin equation. The variable encapsulates the dynamical variables of the surroundings and additionally includes the particles’ size distribution and moment and the applied field’s amplitude and frequency. From an applied perspective, the master variable allows tuning to an optimal MSB biosensing sensitivity range by manipulating both frequency and field amplitude. Calculation of magnetization harmonics in an oscillating applied field is also possible with an approximate closed-form solution in terms of the master variable and a single free parameter.

## Introduction

### Biosensing and nanotechnology

The ability to study the human body non-invasively, both for basic science and medicine, has historically been an excellent marker of scientific progress. From the first clinical X-ray imaging in 1896 [1] to the first human body MRI in 1977 [2], we have now arrived in the era of nanotechnology, allowing researchers to generate many types of nanoscopic sensors that can enter the body themselves and return information with relative non-toxicity. We focus on magnetic nanoparticle biosensors in this paper, and study the magnetic dynamics with the goal to advance the theoretical understanding as well as improve technology.

The magnetization response of Brownian magnetic nanoparticles in an oscillating magnetic field contains extensive information characterizing the microenvironment around the particles. In contrast to Néel rotation where the internal magnetic dipole rotates, Brownian particles rotate mechanically, coupling their motion to their surroundings [3].

Original biosensing schemes with Brownian magnetic nanoparticles used ac susceptibility measurements to quantify the relaxation time of nanoparticles and thereby infer parameters like viscosity of the fluid [4, 5]. Molecular sensing was achieved with magnetorelaxometry [6]. Magnetic resonance imaging has been similarly used [7] and dc SQUID techniques have been used to very sensitively measure relaxation times and bound states [8]. Many other read-out schemes have been developed for measuring nanoparticle magnetizations, including electrochemical sensors [9], plasmonic sensors [10], spin-valve [11, 12], and giant magneto-resistance/-impedance [13, 14]. In particular, spectroscopy of magnetic nanoparticle Brownian motion (MSB) is attractive because of its high sensitivity and *in vivo* applicability [15–17]. Rotational spectroscopy sensing has extended the technique to different regimes [18]. In MSB, scaling arguments have mainly been used to measure variables of interest [19].

### Nonlinear magnetic spectroscopy of Brownian motion (MSB) biosensing with scaling relationships

Nonlinear spectroscopy of the response of Brownian magnetic nanoparticles to applied alternating magnetic fields is a sensitive and practical sensing scheme. The technology, referred to as magnetic spectroscopy of nanoparticle Brownian motion (MSB) has attained a level of sensitivity comparable to many sophisticated molecular detection schemes but has the added advantage of possible *in vivo* molecular detection because low frequency magnetic fields do not harm tissue, iron-oxide nanoparticles are biocompatible, and signal-to-noise is high because biology produces minimal background signal [15, 17].

Nonlinear spectroscopy requires nanoparticles to be excited with a large enough applied field such that the magnetization saturates. The resulting harmonic spectra (or just “harmonics”) of the magnetization conveniently quantify the ability of the particles to physically rotate in time following an applied field. The rotational freedom is affected by many variables (e.g., temperature, viscosity, molecular binding) and scaling relationships have been used extensively to infer these variables from the harmonic spectra. Scaling measurements use the fact that measured harmonics can often be expressed as a function of the product of *pairs* of variables. If one variable is experimentally controllable, an unknown variable can be estimated by modifying the unknown variable and calculating the scaling on the controlled variable that account for the change in harmonics. When the product of variables is identical, magnetic spectra are identical, but when magnetic spectra are not identical, the controlled variable can be scaled, compensating for changes and thus measuring the change in the unknown. The scaling method has been exploited using the field amplitude to temperature ratio to measure temperature [20], and the product of frequency and relaxation time to measure relaxation time [19]. The relaxation time can be a surrogate for a wide variety of biological environmental factors: molecular binding of DNA and cancer biomarkers [3, 15, 17, 21], temperature [22], viscosity [5, 23], and cellular matrix rigidity [24]. Sensing cancer biomarkers *in vivo* with MSB is a truly valuable biomedical possibility. Also a “theranostic” combination of temperature measurements with MSB during magnetic nanoparticle hyperthermia [25] could allow an essential safety mechanism in the simultaneous monitoring of the therapy potency [26].

In this effort we analyze the stochastic Langevin equation that describes Brownian nanoparticle rotations. By non-dimensionalizing the equation we show that it is possible to approximate the dynamics by incorporating the applied field amplitude and frequency with the nanoparticle relaxation time into a single “master variable”. The more general scaling relationships within the master variable compact several previous measurements into general measurements through varying the applied field parameters. The scaling relationship between the field and frequency has pragmatic value when certain dynamics are ideal for an application but certain fields or frequencies are constrained by engineering challenges. For example, the equivalence of applied field and frequency can be exploited when a resonant circuit is constrained by a specific frequency [27] or by biological safety [28]. Additionally, the master variable allows a magnetic spectrometer to be tuned (through field or frequency adjustments) to be maximally sensitive to changes in the environment. Lastly, we demonstrate that a Langevin function using the master variable and a single free parameter can be used to fit harmonic spectra in a closed form approximation.

## Theory

### The Langevin equation for rotation of Brownian magnetic nanoparticles

Brownian magnetic nanoparticles can be used as biosensors because their rotations are exquisitely affected by their surroundings. Brownian particles rotate mechanically in solution under the presence of an oscillating applied field, distinguishing themselves from those magnetic nanoparticles that rotate their moment through internal restructuring of electronic states as in Néel rotation. Néel particles are indeed affected by local temperature, but are not coupled to the suspension viscosity or chemical bonds on the exterior of the particle, limiting their applicability as biosensors. We henceforth restrict our discussion to Brownian particles.

The rotational dynamics of ensembles of Brownian magnetic nanoparticles have previously been studied using Langevin equation approaches [29–35]. If the magnetic axis of a nanoparticle is spatially fixed to an axis of the particle as in larger magnetic nanoparticles with large anisotropy, the nanoparticles can be described as an ensemble of magnetic dipoles. When suspended in a solution and immersed in a magnetic field, the particle’s magnetization vector **m** aligns to the field but is slowed by viscous torques. A statistical distribution of alignments occurs at finite temperature. Dynamics of magnetic nanoparticles can thus be modeled phenomenologically with a Langevin equation, a differential equation for the magnetization vector that includes stochastic torques.

The rotational Reynolds Re number depends on the suspending fluid’s dynamic viscosity *η* and density *ρ*, and a particle’s hydrodynamic diameter *d* and rotational velocity—approximated to be the frequency of rotation multiplied by the particle’s hydrodynamic diameter. For magnetic nanoparticle biosensing applications, an order of magnitude estimation with *ρ* = 10^3^ kg/m^3^, *f* = 1 kHz, *d* = 100 nm, and *η* = 10^−3^ kg/(m⋅s) leads to *Re* = *ρfd*^2^/*η* ≈ 10^−5^ so that angular accelerations are negligible.

Neglecting angular accelerations simplifies the Langevin equation to be first order in time. The dynamics of a magnetic particle rotating in a fluid depends on the particle’s hydrodynamic volume *V* = *πd*^3^/6 and magnetic moment magnitude *μ*. The fluid’s temperature *T* and viscosity *η* also impact the rotations [29, 34, 35].

$$\begin{array}{c} \frac{dm}{dt}=\frac{1}{6\eta V}\left(\mu m\times H_{t}-\theta_{t}\right)\times m. \end{array}$$ (1)

A time-varying applied magnetic field **H**_*t*_ changes the dynamics and is supplemented by a stochastic torque ***θ***_*t*_ that accounts for collisions with fluid molecules. Because this torque is due to many collisions, it can be modeled as a normally distributed, or Gaussian, white noise torque proportional to the Einstein-Smoluchowski diffusion constant *D* = 6*ηV k*_B_*T* [31, 34, 36, 37]. Using the white noise process **λ**_*t*_, we have $\theta_{t}=\sqrt{2D}\lambda_{t}$. The white noise process is mathematically expressed as a vector of zero mean, delta-autocorrelated in time (i.e., Markovian) separate white noise processes so that for *i*, *j* ∈ *x*, *y*, *z* we have

$$\begin{array}{c} \langle \lambda_{t}\rangle =0,\;\langle \lambda_{t}\lambda_{s}\rangle =\delta_{ij}\delta \left(t-s\right). \end{array}$$ (2)

### Non-dimensionalizing the dynamical Langevin equation

Writing the white noise torque in Eq 1 in terms of the white noise process, and multiplying both sides by *k*_B_ *T*, the Langevin equation can be rewritten in terms of two commonly used variables [38, 39]: the unitless field ***ξ***_*t*_ = *μ* **H**_*t*_/*k*_B_*T* and the zero-field Brownian relaxation time *τ*_B_ = 3*ηV*/*k*_B_*T*, so that we have

$$\begin{array}{c} \frac{dm}{dt}=\frac{\left(m\times \xi_{t}\right)\times m}{2\tau_{B}}+\frac{m\times \lambda_{t}}{\sqrt{\tau_{B}}}. \end{array}$$ (3)

Note that we see from Eq 2 that the white noise process has dimensions of $\sqrt{1/t}$ so the term containing the square root of the relaxation time in Eq 3 is dimensionally correct.

The intervals of the Wiener process are Gaussian random variables proportional to the square root of the time interval, $dW_{t}∼N_{t}\left(0,1\right)\sqrt{dt}$ where **N**_*t*_(0, 1) is a 3-vector of Gaussian random variables each with mean zero and unit variance [39, 40]. Therefore, the solution to the stochastic differential equation is

$$\begin{array}{c} m=\int \frac{\left(m\times \xi_{t}\right)\times m}{2\tau_{B}}dt+\int \frac{m\times N_{t}\left(0,1\right)}{\sqrt{\tau_{B}}}\sqrt{dt}, \end{array}$$ (4)

an equation that is not in general solvable due to the multiplicative noise and nonlinear dynamics involved.

Many of the applications of magnetic nanoparticles employ oscillating magnetic fields. We simulate the field with $H_{t}=H_{0}\cos\left(2\pi ft\right)\hat{z}$ with amplitude *H*_0_ and frequency *f*. In unitless form, $\xi_{t}=\xi_{0}\cos2\pi ft\hat{z}$. We can also non-dimensionalize the timescale by employing the transformation t* = *tf* so time runs from 0 → 1 in a single period of the oscillating field. Scaling laws have been shown previously in the corresponding Fokker-Planck equation [19, 33]. For example, the magnetic dynamics are exactly dependent on the quantity *fτ*_B_ which we henceforth call the unitless frequency Ω [33].

The solution to the stochastic differential equation in dimensionless form is

$$\begin{array}{c} m=\frac{\xi_{0}}{2\Omega }\int \left(m\times \cos2\pi t^{*}\hat{z}\right)\times m\;dt^{*}+\int \frac{m\times N_{t}\left(0,1\right)\sqrt{dt^{*}}}{\sqrt{\Omega }} \end{array}$$ (5)

using the three dimensionless variables

$$\begin{array}{c} \Omega =f\tau_{B}\;t^{*}=tf\;\xi_{0}=\frac{\mu H_{0}}{k_{B}T}. \end{array}$$ (6)

### Scaling law with the master variable

In the present work, we develop an approximate scaling law combining the unitless field amplitude and unitless frequency inspired by the observation that the parameter in front of the first integral in Eq 5 determines much of the dynamics. We now define the ratio

$$\begin{array}{c} A=\frac{\xi_{0}}{\Omega } \end{array}$$ (7)

and refer to it as the “master variable”.

In the case where *ξ*_0_ > Ω, the stochastic term has less impact than the deterministic term and the dynamics are completely parameterized by A. This range should be feasible for the hundred-microsecond relaxation times and single kHz frequencies as well as the 100 nm diameter particles and 10–20 mT field strengths typically used in MSB experiments [15, 19, 22, 24].

Ignoring the stochastic torque entirely means the dynamics are exactly and completely parameterized by the master variable. The method of ignoring the stochastic term has been used (see the magneto-dynamics approximation in Ref. [41]) and leads to

$$\begin{array}{c} m\approx A\int \left(m\times \cos2\pi t^{*}\hat{z}\right)\times m\;dt^{*}, \end{array}$$ (8)

making $m(t^{*},A)$ only. We use Eq 7 to interpret Eq 8. Increasing the frequency leads to dynamics equivalent to dynamics with a lower field amplitude. For example, the scaling relationship explains an intuitive relationship: nanoparticles rotating in a stronger field align faster and thus are able to follow that field more closely (rotate in phase). Equivalently, particles exposed to a lower frequency field have more time to align before the field changes its sign, therefore remaining more in phase. That this scaling relationship is actually linear proportionality (i.e., scaling field and frequency by the same factor admits the same dynamics), as opposed to some other functional relationship, is not immediately intuitive, but is mathematically justified using the master variable.

## Methods

### Simulations of biosensing applications confirm the master variable scaling approximation

We solve the stochastic differential equation (Eq 3) with a numerical expression of Eq 4 using Heun’s scheme [40, 42]. The two-step Heun solver has predictor $\bar{m}\left(t^{*}+\Delta t^{*}\right)=\bar{m}\left(t^{*}\right)+\Delta \bar{m}$ defined by

$$\begin{array}{c} \Delta \bar{m}=\frac{\xi_{0}\Delta t^{*}}{2\Omega }\left(m\times \cos2\pi t^{*}\hat{z}\right)\times m+m\times N_{t^{*}}\left(0,1\right)\sqrt{\frac{\Delta t^{*}}{\Omega }} \end{array}$$ (9)

and true magnetization is then defined by

$$\begin{array}{c} m\left(t^{*}+\Delta t^{*}\right)=m\left(t^{*}\right)+\frac{\xi_{0}\Delta t^{*}}{4\Omega }\left[\left(\bar{m}\times \cos2\pi (t^{*}+\Delta t^{*})\hat{z}\right)\times \bar{m}\right.\\ +\left.\left(m\times \cos2\pi t^{*}\hat{z}\right)\times m\right]+\frac{1}{2}\left(m+\bar{m}\right)\times N_{t^{*}}\left(0,1\right)\sqrt{\frac{\Delta t^{*}}{\Omega }} \end{array}$$ (10)

where **N**_*t*_(0, 1) is a 3-vector of Gaussian-distributed random numbers each with mean zero and unit standard deviation.

The time steps must be sufficiently small to faithfully capture the full rotational dynamics of the particles. Inspired by other works [31, 41], we restrict the time step to be a small fraction of the relaxational timescale and frequency product; specifically Δt* < 0.01Ω. When magnetic fields are used, the timescales are shorter but for our ranges we did not see numerical issues. For works aiming to extend this model to add additional physics, like interactions between particles, it would be necessary to calculate all existing timescales and appears sufficient to keep time steps to 1% of the shortest timescale.

All simulations employ 10^5^ particles, meaning that the ultimate solution is the average of 10^5^ solutions of the stochastic equation. As is typical for magnetic particles, the radii (both core and hydrodynamic) are assumed to be lognormally distributed [33, 43–45]. The probability of having a particle with a radius *r* is then

$$\begin{array}{c} p\left(r\right)=\frac{1}{\sqrt{2\pi }}\frac{1}{r\sigma_{r}}\exp\left[-\frac{{\left\{\ln\left(\frac{r}{m_{r}}\sqrt{1+\frac{s_{r}^{2}}{m_{r}^{2}}}\right)\right\}}^{2}}{2\sigma_{r}^{2}}\right] \end{array}$$ (11)

where the mean and standard deviation of the distribution are *m*_*r*_ and *s*_*r*_ respectively, and the scale parameter is defined $\sigma_{r}=\sqrt{\ln(1+\frac{s_{r}^{2}}{m_{r}^{2}})}$. We estimate that standard deviations are 10% of the mean.

In stochastic simulations, we solve for the mean magnetization using nanoparticles with probabilistically distributed radii. The variability is expressed both in the unitless field through the magnetic moment (a function of the core volume) as well as in the unitless frequency through the relaxation time (a function of the hydrodynamic volume). Even more information can be included within the master variable by defining the master variable of the mean parameters

$$\begin{array}{c} A=\frac{\langle \xi_{0}\rangle }{\langle \Omega \rangle }. \end{array}$$ (12)

In discussion of realistic experiments, the master variable can then be written in terms of the mean values of particles with size distributions.

In magnetic nanoparticle spectroscopy or MSB, the derivative harmonics of the magnetization are used as a metric to analyze the dynamics [15, 23, 27]. The *l*-th derivative harmonic is the Fourier transformation of the magnetization parallel to the applied field $F_{l}\left(m_{z}\right)=\int m_{z}\exp\left(il\omega t\right)dt$ at integer multiple *l* of the fundamental frequency *ω* = 2*πf*. We henceforth refer to the *l*-th derivative harmonic as $a_{l}=lF_{l}\left(m_{z}\right)$ where the factor of the harmonic number accounts for the derivative.

## Results

### Simulations and experimental validation of scaling laws in magnetization and magnetization harmonics

**Fig 1:**
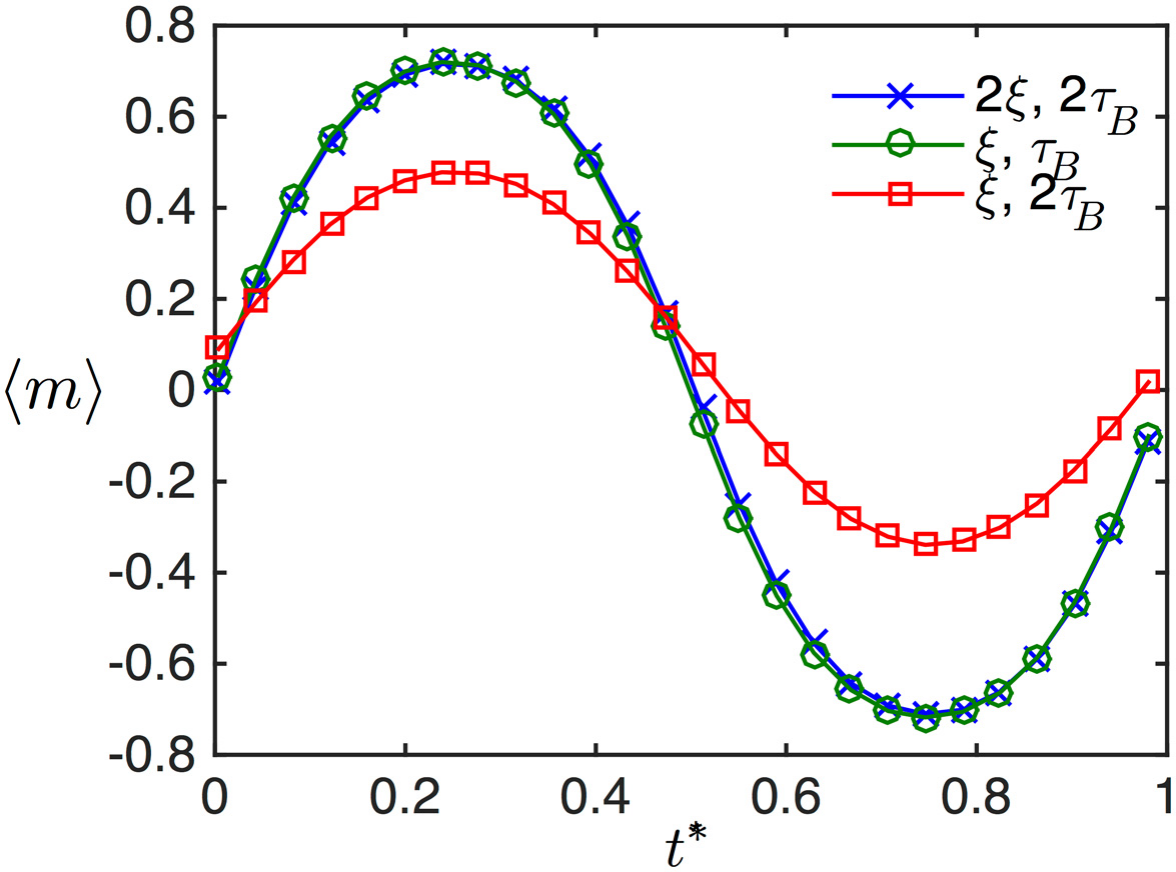
(Color online) The scaling relationship between the magnetic field and relaxation time is demonstrated by simulating the mean magnetization response of a polydisperse ensemble of magnetic nanoparticles in an oscillating applied magnetic field. The normalized mean magnetization is identical if the field and the relaxation time are multiplied by the same number.

The master variable scaling is simulated by plotting the average magnetization over time at two relaxation times and two unitless fields in Fig 1. Here, because 〈*ξ*_0_〉 > 〈Ω〉, the scaling relationship between the field and relaxation time is an excellent approximation. It is visible by eye that when the relaxation time is doubled, saturation decreases, yet when the applied field strength (or the moment) is also doubled, the initial dynamics are restored. This is an example of the generalized scaling relationship shown in Eq 7.

**Fig 2:**
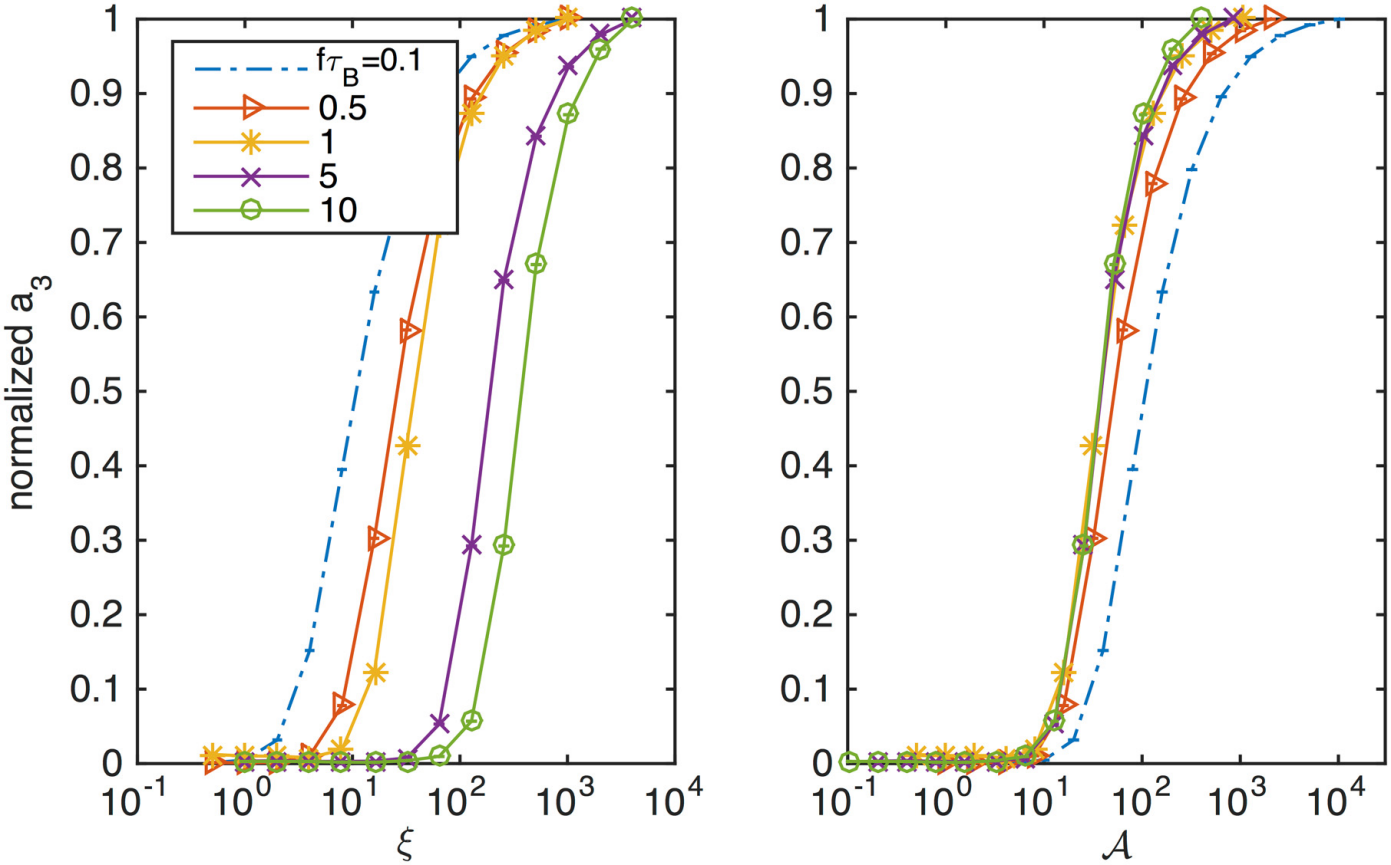
(Color online) The scaling relationship in the magnetization harmonics is simulated by varying the mean unitless field. The normalized third harmonic plotted against the mean magnetic field amplitudes in the left panel, and against the master variable on the right. The plots illustrate that spectra from nanoparticles with different relaxation times (when 〈Ω〉 > 1) will align when plotted against their respective master variable.

**Fig 3:**
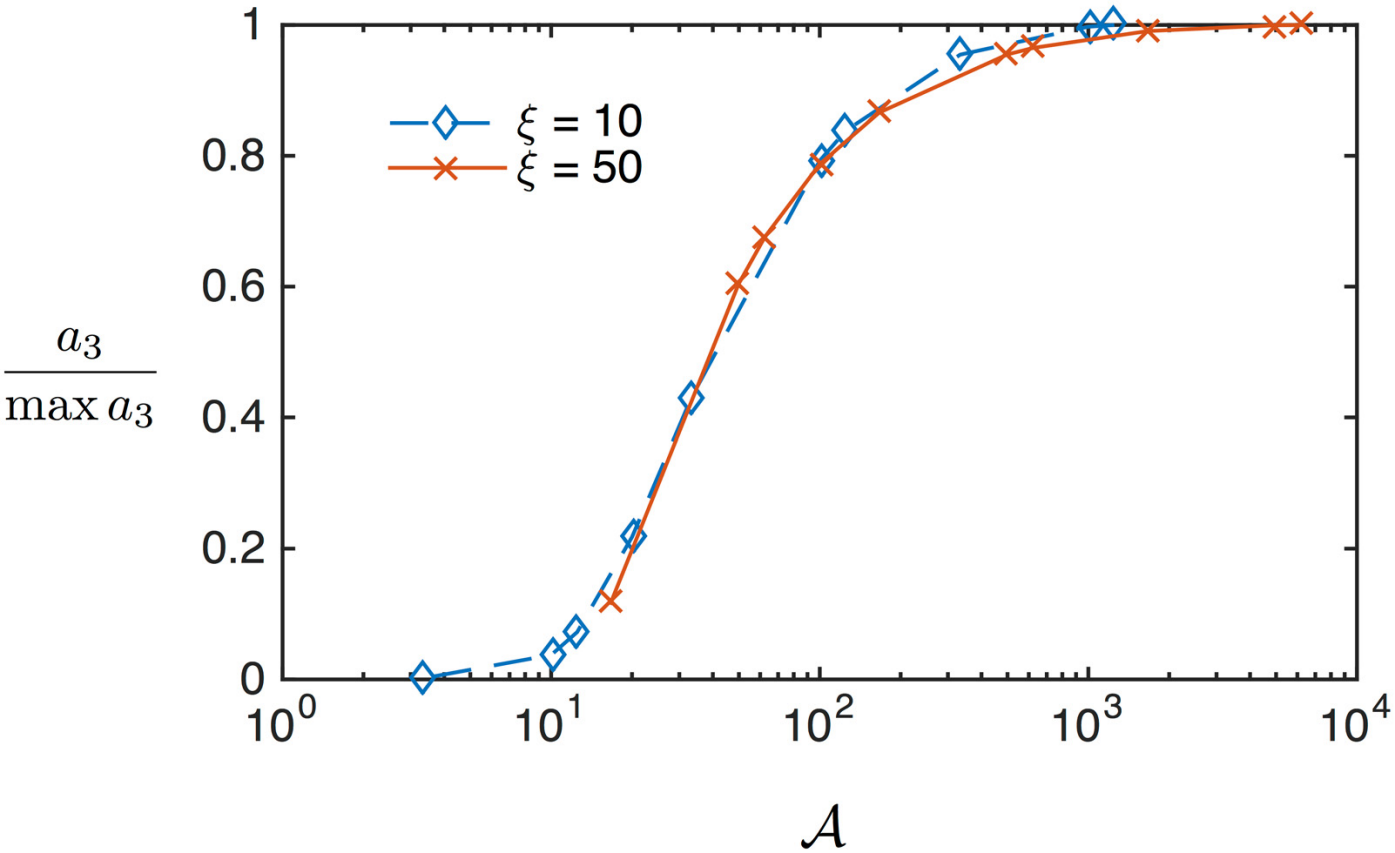
(Color online) Scaling relationships in the magnetization harmonics are simulated by varying the mean relaxation time and plotting against the master variable. The normalized third harmonic is equivalent for two values of the unitless field.

The scaling relationship between the field and the frequency or relaxation time also is evident in the harmonics. Fig 2 shows the normalized third harmonic *a*_3_/ max *a*_3_ with respect to the unitless field amplitude in the left panel, and the same data plotted against the master variable in the right panel. The several curves are all made at different mean relaxation times and the same frequency. The harmonics are unchanged by scaling the relaxation time when plotted against the master variable when *f*〈*τ*_B_〉 = 〈Ω〉 > 1. This is a different constraint on Ω related to saturation effects and the specific field amplitude chosen. Equivalently put, when a change in relaxation time is accounted for by an identical change in field amplitude, the harmonic spectra are identical. The data in Fig 2 are from simulations where 〈*ξ*_0_〉 was varied for five different values of 〈Ω〉, but the same scaling is also simulated by varying 〈*τ*_B_〉 for two different values of 〈*ξ*_0_〉 in Fig 3.

**Fig 4:**
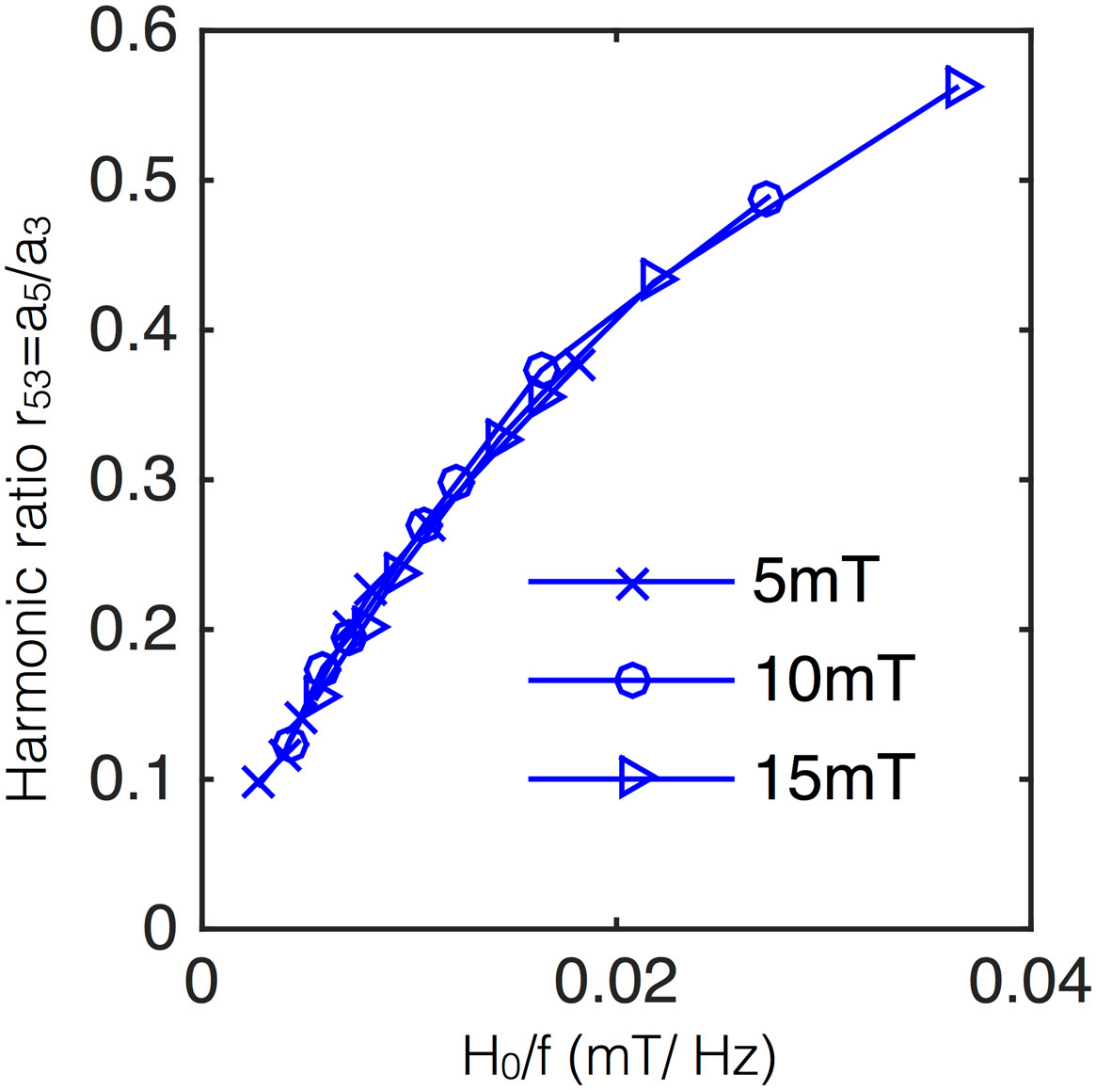
The approximate scaling relationship between field amplitude and frequency is demonstrated experimentally. The ratio of the fifth to third harmonic *r*_53_ = *a*_5_/*a*_3_ from the same nanoparticles at different oscillating field amplitudes ranging from 5–15 mT is plotted against the ratio of the field amplitude to the frequency. The spectra are equivalent, illustrating the scaling law.

Using the apparatus and nanoparticle detailed in Refs. [22, 46] we provide a preliminary validation of the scaling law in the range typically used for MSB. Micromod Partikeltechnologie iron oxide nanoparticles with hydroxyethyl-starch coatings and 100 nm hydrodynamic diameters were studied at three different magnetic field amplitudes and the same frequencies for each field (400-2,000 Hz). The experiment validates the generalized scaling: when the data is plotted against the ratio of the field to frequency, the ratio of the fifth to third harmonic is equivalent. These data are shown in Fig 4. The scaling works because these particles have mean Brownian relaxation time 〈*τ*_B_〉 ∼ 0.5 ms. Even at 400 Hz frequencies where 〈Ω〉 ∼ 0.2, it appears the scaling is a reasonable approximation as *ξ*_0_ > Ω but not large enough to distort with saturation effects.

### The master variable is not directly temperature-dependent

**Fig 5:**
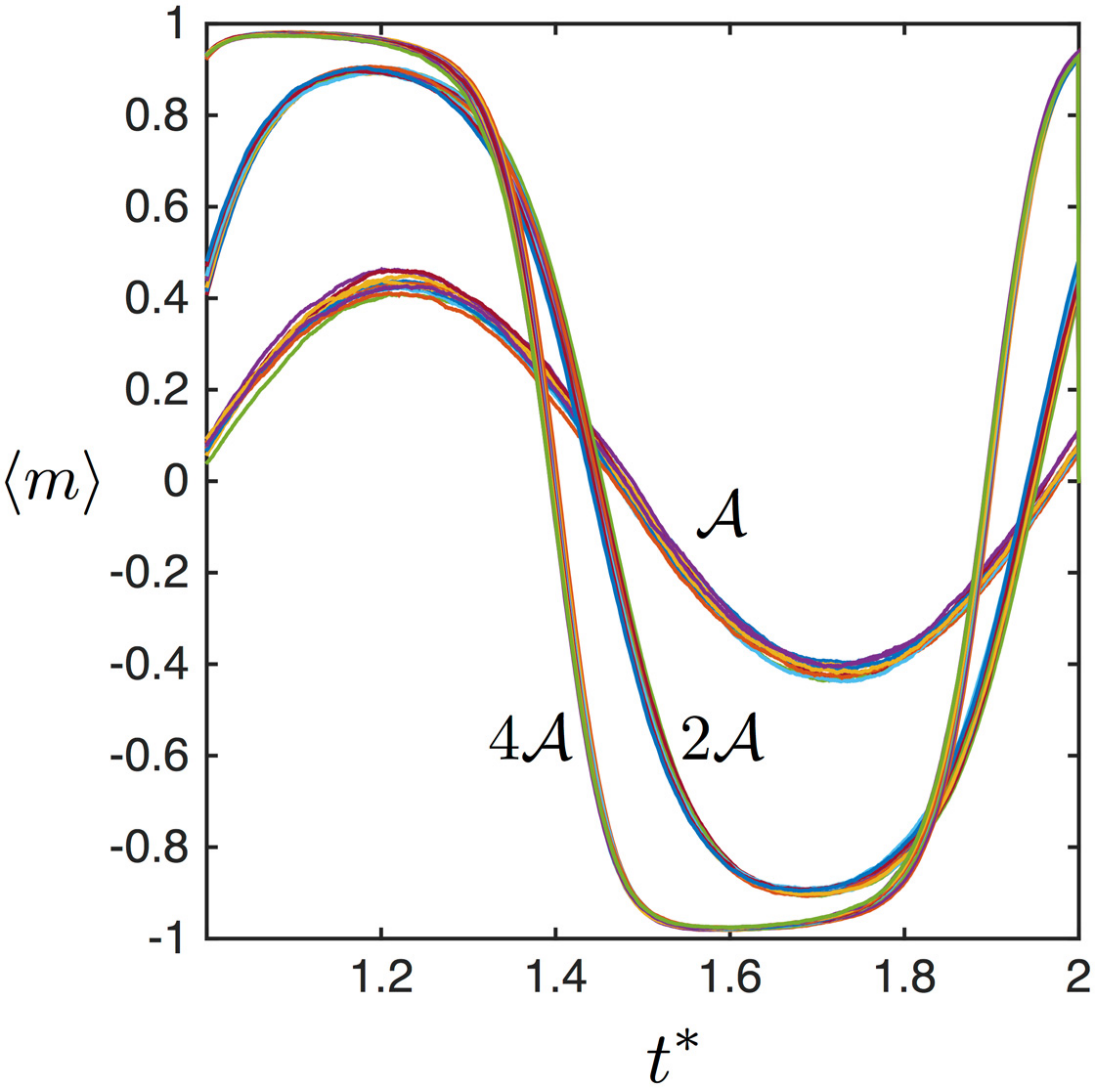
(Color online) Various values of the master variable lead to different dynamics. For each value of the master variable, ten different magnetizations, ranging by 10 K from 250–350 K, are plotted in order to demonstrate the stability to realistic temperature variations.

Because the master variable does not directly depend on temperature (it divides out of the ratio of the unitless field over the relaxation time), changing temperature at some value of the master variable should not change the mean dynamics. In reality the master variable will contain an indirect temperature dependence due to the dependence of the viscosity on the temperature but we do not consider that effect here. Fig 5 shows simulations of the mean magnetization are stable to temperature perturbations (that is, changes exclusively in the stochastic term of the Langevin equation). In Fig 5, different values of the master variable (A,2A,4A) result in obviously different magnetization dynamics. For each fixed value of the master variable, the simulations were completed at ten different temperatures (250 K to 350 K by 10 K increments). The mean magnetizations are indistinguishable at different temperatures. However, it is noticeable that fluctuations about the mean are larger at lower master variables. Indeed, the scaling relationships for the higher moments behave differently than the first moment.

### Connection with ac susceptibility

Measurement of the relaxation time for magnetic nanoparticle biosensing can be achieved using one-dimensional ac susceptibility [4]. For a small oscillating applied field as typical in ac susceptibility measurements, the magnetization parallel to the applied field (as above we specify $\xi =\xi_{0}\cos2\pi t^{*}\hat{z}$) can be written with the Debye equation [35, 47, 48],

$$\begin{array}{c} m_{z}=\frac{\xi_{0}}{3}\left(\frac{\cos2\pi t^{*}}{1+{\left(2\pi \Omega \right)}^{2}}+\frac{2\pi \Omega \sin2\pi t^{*}}{1+{\left(2\pi \Omega \right)}^{2}}\right) \end{array}$$ (13)

so that when Ω > 1, the Debye magnetization reduces to

$$\begin{array}{c} m_{z}\approx \frac{A}{6\pi }\sin2\pi t^{*} \end{array}$$ (14)

and the dynamics are again completely described by the master variable.

It is worthwhile to note that in the opposite limit (Ω < 1), we can use the binomial theorem to expand the denominators as

$$\begin{array}{c} m_{z}\approx \frac{\xi_{0}}{3}\left(1+4\pi \Omega \right)\left[\cos2\pi t^{*}+2\pi \Omega \sin2\pi t^{*}\right], \end{array}$$ (15)

so ignoring terms of higher order than linear (i.e., keeping up to O(Ω)) we arrive at

$$\begin{array}{c} m_{z}\approx \frac{\xi_{0}}{3}\left[\left(1+4\pi \Omega \right)\cos2\pi t^{*}+2\pi \Omega \sin2\pi t^{*}\right]. \end{array}$$ (16)

Because Ω < 1, we expect the dynamics to mostly be determined by the term $m_{z}\approx \frac{\xi_{0}}{3}\cos2\pi t^{*}$, but the next-highest order term is due to the product of the unitless field with the unitless frequency. This term may explain the findings of Shah et al. who suggest that this factor of the field multiplied by the frequency (the “slew rate”) determines the dynamics [49].

## Discussion and applications of the master variable

### Biosensing using the generalized scaling relationship

In MSB, typical nanoparticles have 20 nm core and 50 nm hydrodynamic radii [15, 22]. These particles ensure Brownian rotation so that the rotational dynamics are coupled with the environment. In water having viscosity 1 mPa-s at room temperature, these particles have mean Brownian relaxation time 〈*τ*_B_〉 ∼ 0.5 ms and some in the distribution will be much above this value. That means that with fields at or above 1 kHz frequencies, 〈Ω〉 > 0.5. With typical particle sizes, saturation magnetizations of roughly 250 kA/m and applied fields of 5 mT/*μ*_0_ make the unitless field *ξ*_0_ ∼ 6 and thus at a single kHz or below, A>12. These parameters are typical for biosensing with MSB [15, 22, 27], justifying the generalized scaling in that context (see Fig 4).

In many cases, magnetic spectroscopy of Brownian motion (MSB) has employed single scaling relationships (between field amplitude and temperature [20], and frequency and relaxation time [19]). The master variable combines all of these scaling relationships into a generalized form, thus allowing scaling of field to measure relaxation time (and all the parameters for which relaxation time is a surrogate: viscosity, temperature, binding, etc.). The ability to scale field against relaxation time was shown in simulation in Fig 1 and experimentally in Fig 4. In the related context of rotational spectroscopy, the ability to rescale the field to optimally sense at a different frequency was demonstrated [50].

**Fig 6:**
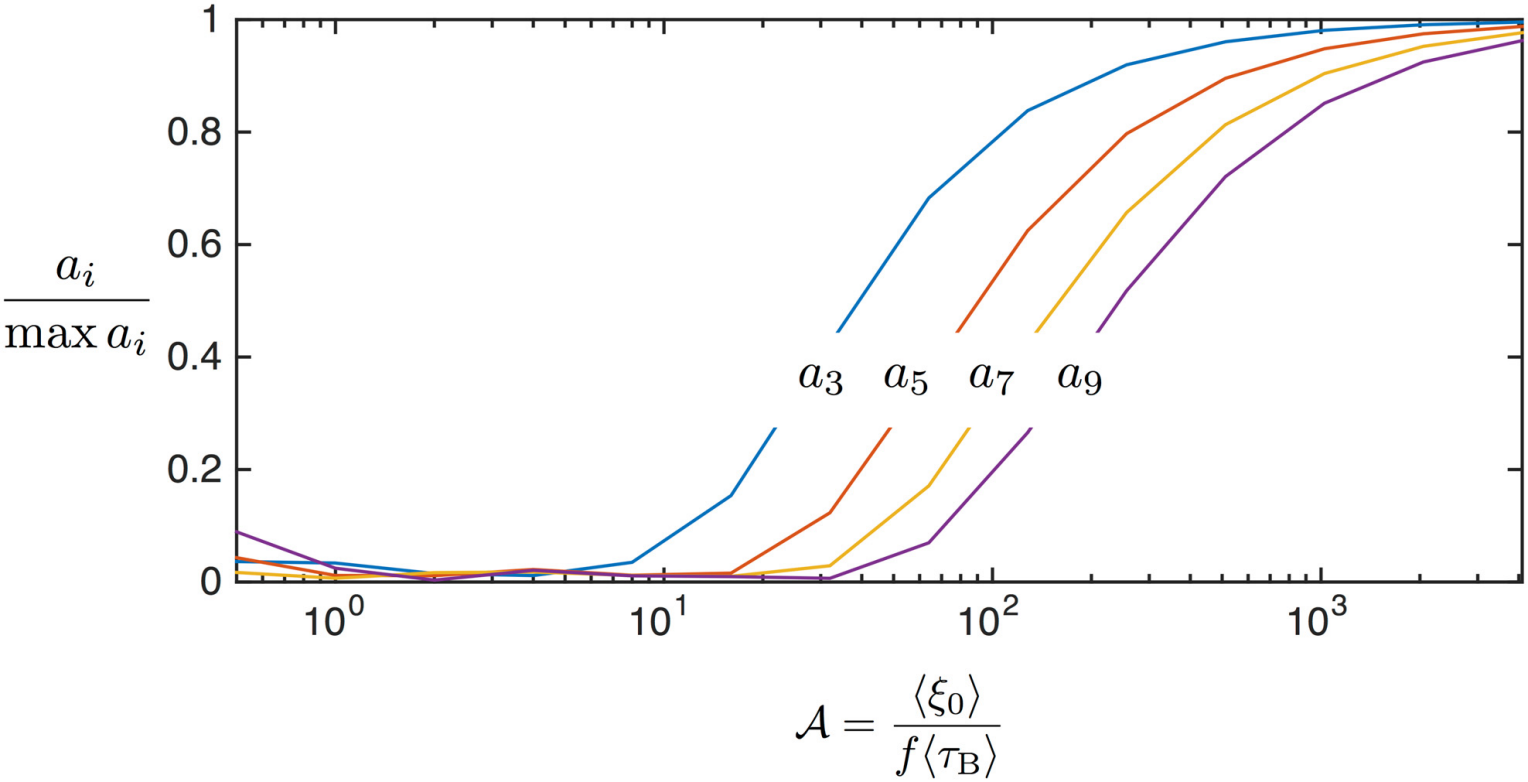
(Color online) Simulated magnetization harmonics are shown for values of A. The shapes of the curves are sigmoidal, showing that as the field amplitude is increased the magnetization saturation increases, and each harmonic saturates. The region of each harmonic containing the steepest slope provides the largest changes in the harmonic for small changes in the master variable time, i.e., the optimal sensitivity range for MSB biosensing.

Simulated harmonic spectra are shown in Fig 6 as the mean unitless field is increased. The curves are sigmoidal, indicating that all harmonics are zero when a low amplitude field is applied—i.e., the magnetization responds linearly. In a higher amplitude field, higher harmonics arise as the magnetization response becomes nonlinear. At some large field amplitude (*ξ*_0_ ∼ 10^3^) each of the harmonics saturates and the magnetization response approaches a square wave.

Each harmonic has a steepest slope for some value of the master variable. The value that produces the steepest slope is important because it indicates the largest change in the harmonics for a small change in the master variable. The master variable includes the relaxation time, a typical parameter measured as a surrogate for binding [4, 15]. Therefore, there is an optimal value of the master variable that provides the highest sensitivity to changes in relaxation time. Given the equivalence of field and frequency in the master variable, the optimal sensing can be achieved for nanoparticles with a given relaxation time by adjusting field amplitude, frequency, or both. For typical Brownian nanoparticles the optimal value occurs for the third harmonic in the range of A∼25 roughly 25 mT/*μ*_0_ at 1 kHz, well within typical MSB biosensing ranges [22, 27].

### Phenomenological closed form approximation of harmonics using a Langevin function of the master variable

Having established that magnetization can be expressed as a function of a master variable we were then curious what functional form, if any, the harmonics followed in terms of the master variable. As field amplitudes or frequencies are varied, we see sigmoidal shapes for the harmonics (see Fig 6 for example). A familiar function in magnetism, the Langevin function, proves to be a good approximation of the sigmoidal shapes when the argument is the master variable with a computationally determined scale factor for each harmonic.

**Fig 7:**
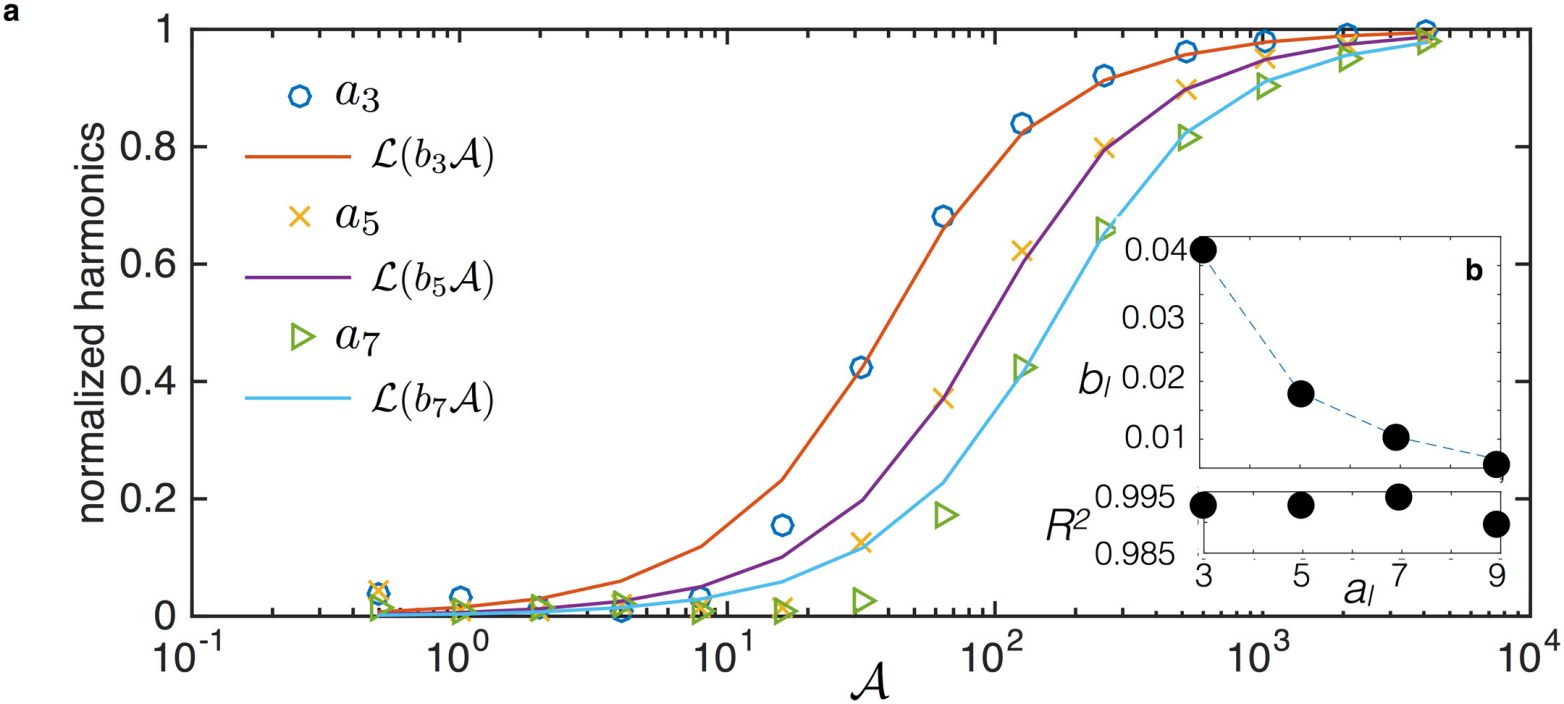
(Color online) a) Simulated normalized harmonics (data points) over a range of the master variable A are fit by a domain-scaled Langevin function (lines). b) Inset are the values of the computational fitting parameters for each harmonic *b*_*l*_ and the associated *R*^2^ > 0.98 values indicating the strong fit.

The simulated harmonics and the domain scaled Langevin function for each harmonic are plotted against the master variable in Fig 7. The computationally determined parameter is called *b*_*l*_, such that the lines are described by $a_{l}=L\left(b_{l}A\right)=\coth\left(b_{l}A\right)-1/\left(b_{l}A\right)$. The values of *b*_*l*_ and the *R*^2^ values are plotted to show the strong fit. Justification of this approximation is not developed theoretically, but should be considered in future works.

In general, because of the shape of the Langevin function the inflection point of the data cannot be reconstructed and the lower values of A are less accurate. The utility of the approximation is due to the compression of the multi-parameter space into the single master variable when using the master variable to include varying relaxation times, frequencies, and field amplitudes. The domain-scaled Langevin function contrasts other models for the harmonics, for example, the logistic function that needs many free parameters [18, 51]. The simple form of the approximation is surprising because typical sigmoidal curve fits require several free parameters [51].

## Conclusions

We showed by non-dimensionalizing the Langevin equation that, in the regime of large unitless field compared to relaxation time oscillating field period (*ξ*_0_ > Ω), the dynamics of Brownian magnetic nanoparticles are determined by a single master variable, A, that incorporates most typically considered nanoparticle properties (including size distributions) as well as applied magnetic field properties. The master variable parameterization of the dynamics was confirmed by numerically solving the Langevin equation and in experiment. Conceptually, the master variable indicates that the field to frequency ratio controls the dynamics, and that scaling one can be accounted for by scaling the other identically. (In some regimes the product may also be meaningful—see the discussion of ac susceptibility.)

Then, in the context of biosensing with nonlinear Brownian magnetic nanoparticle spectroscopy (MSB), using the master variable provides generalized scaling relationships. The scaling relationships show that it is possible to measure changes in the relaxation time by changing the magnetic field amplitude or the frequency. The scaling can be interpreted to explain an intuitive equivalence between magnetic field amplitude and frequency. For example, stronger fields align particles faster and thus phase lagging is decreased in an equivalent way that lower frequencies allow particles more time to rotate and thereby remain in phase.

We briefly illustrated an optimal sensitivity range to changes in the relaxation time, and thus optimal biosensing performance, can be achieved by using a specific value of the master variable, but this must be demonstrated experimentally moving forward. The equivalence of field and frequency is particularly applicable when this optimal MSB range is desired, yet some applied field properties are practically constrained.

The master variable encapsulates many parameters required to describe particle dynamics and is thereby valuable in a phenomenological fit to the harmonics. The closed-form approximation is a Langevin function of the master variable scaled by a numerically fit parameter at each harmonic. The fit is very good, especially at higher values of the master variable. Other sigmoidal forms have been used to fit harmonic data, but the advantage to this approach lies in the single requisite free parameter: the domain-scaling factor at a specific harmonic.

Many biomedical applications can benefit from magnetic nanoparticle biosensors. Sensitive assays for specific molecules should soon be possible *in vivo*, and the ability to infer environments surrounding nanoparticles may be essential to measuring the temperature and ensuring safety during proposed magnetic nanoparticle hyperthermia cancer therapy. These advances are sure steps toward implementation of nanotechnology in modern medicine.

## References

[1] Frost E. Experiments on the X-rays. Science. 1896; p. 235–236. 10.1126/science.3.59.235 17769968

[2] Damadian R, Goldsmith M, Minkoff L. NMR in cancer: XVI. FONAR image of the live human body. Physiological Chemistry and Physics. 1976;9(1):97–100. 909957

[3] Rauwerdink A, Weaver J. Measurement of molecular binding using the Brownian motion of magnetic nanoparticle probes. Applied Physics Letters. 2010;96(3):033702 10.1063/1.3291063

[4] Chung S, Hoffmann A, Bader S, Liu C, Kay B, Makowski L, et al Biological sensors based on Brownian relaxation of magnetic nanoparticles. Applied Physics Letters. 2004;85(14):2971–2973. 10.1063/1.1801687

[5] Calero-DdelC V, Santiago-Quiñonez D, Rinaldi C. Quantitative nanoscale viscosity measurements using magnetic nanoparticles and SQUID AC susceptibility measurements. Soft Matter. 2011;7(9):4497–4503. 10.1039/c0sm00902d

[6] Eberbeck D, Bergemann C, Wiekhorst F, Steinhoff U, Trahms L. Quantification of specific bindings of biomolecules by magnetorelaxometry. J Nanobiotechnol. 2008;6(4):13. 10.1186/1477-3155-6-4 PMC2374834 18334023

[7] Perez J, Josephson L, O’Loughlin T, Högemann D, Weissleder R. Magnetic relaxation switches capable of sensing molecular interactions. Nature Biotechnology. 2002;20(8):816–820. 10.1038/nbt720 12134166

[8] Chemla Y, Grossman H, Poon Y, McDermott R, Stevens R, Alper M, et al Ultrasensitive magnetic biosensor for homogeneous immunoassay. Proceedings of the National Academy of Sciences. 2000;97(26):14268–14272. 10.1073/pnas.97.26.14268 PMC18907 11121032

[9] Pan J, Yang Q. Antibody-functionalized magnetic nanoparticles for the detection of carcinoembryonic antigen using a flow-injection electrochemical device. Analytical and Bioanalytical Chemistry. 2007;388(1):279–286. 10.1007/s00216-007-1224-0 17393156

[10] Anker J, Hall W, Lyandres O, Shah N, Zhao J, Van Duyne R. Biosensing with plasmonic nanosensors. Nature Materials. 2008;7(6):442–453. 10.1038/nmat2162 18497851

[11] Ferreira H, Graham D, Freitas P, Cabral J. Biodetection using magnetically labeled biomolecules and arrays of spin valve sensors. Journal of Applied Physics. 2003;93(10):7281–7286. 10.1063/1.1544449

[12] Li G, Sun S, Wilson RJ, White RL, Pourmand N, Wang SX. Spin valve sensors for ultrasensitive detection of superparamagnetic nanoparticles for biological applications. Sensors and Actuators A: Physical. 2006;126(1):98–106. 10.1016/j.sna.2005.10.001 18414592 PMC2293286

[13] Chiriac H, Herea DD, Corodeanu S. Microwire array for giant magneto-impedance detection of magnetic particles for biosensor prototype. Journal of Magnetism and Magnetic Materials. 2007;311(1):425–428. 10.1016/j.jmmm.2006.11.207

[14] Koets M, Van der Wijk T, Van Eemeren J, Van Amerongen A, Prins M. Rapid DNA multi-analyte immunoassay on a magneto-resistance biosensor. Biosensors and Bioelectronics. 2009;24(7):1893–1898. 10.1016/j.bios.2008.09.023 19028086

[15] Zhang X, Reeves D, Perreard I, Kett W, Griswold K, Gimi B, et al Molecular sensing with magnetic nanoparticles using magnetic spectroscopy of nanoparticle Brownian motion. Biosensors and Bioelectronics. 2013;50:441–446. 10.1016/j.bios.2013.06.049 23896525 PMC3844855

[16] Rauwerdink A, Weaver J. Concurrent quantification of multiple nanoparticle bound states. Medical Physics. 2011;38(3):1136–1140. 10.1118/1.3549762 21520825 PMC3055696

[17] Zhang X, Reeves D, Shi Y, Gimi B, Nemani K, Perreard I, et al Toward Localized In Vivo Biomarker Concentration Measurements. Magnetics, IEEE Transactions on. 2015;51(2):1–4. 10.1109/TMAG.2014.2324993 PMC4507828 26203196

[18] Dieckhoff J, Lak A, Schilling M, Ludwig F. Protein detection with magnetic nanoparticles in a rotating magnetic field. Journal of Applied Physics. 2014;115(2):024701 10.1063/1.4861032

[19] Weaver J, Kuehlert E. Measurement of magnetic nanoparticle relaxation time. Medical Physics. 2012;39(5):2765–2770. 10.1118/1.3701775 22559648 PMC3350541

[20] Weaver J, Rauwerdink A, Hansen E. Magnetic nanoparticle temperature estimation. Medical Physics. 2009;36:1822 10.1118/1.3106342 19544801 PMC4109636

[21] Weaver J, Rauwerdink A. Quantitation of nanoparticle concentrations in microscopic bound states. Medical Physics. 2010;37:358 10.1118/1.3469126

[22] Perreard I, Reeves D, Zhang X, Kuehlert E, Forauer E, Weaver J. Temperature of the magnetic nanoparticle microenvironment: estimation from relaxation times. Physics in Medicine and Biology. 2014;59(5):1109 10.1088/0031-9155/59/5/1109 24556943 PMC4021595

[23] Rauwerdink A, Weaver J. Viscous effects on nanoparticle magnetization harmonics. Journal of Magnetism and Magnetic Materials. 2010;322(6):609–613. 10.1016/j.jmmm.2009.10.024

[24] Weaver J, Rauwerdink K, Rauwerdink A, Perreard I. Magnetic spectroscopy of nanoparticle Brownian motion measurement of microenvironment matrix rigidity. Biomedical Engineering. 2013;58(6):547–550. 23945110 10.1515/bmt-2013-0012

[25] Jordan A, Scholz R, Wust P, Fähling H, Felix R. Magnetic fluid hyperthermia (MFH): Cancer treatment with AC magnetic field induced excitation of biocompatible superparamagnetic nanoparticles. Journal of Magnetism and Magnetic Materials. 1999;201(1):413–419. 10.1016/S0304-8853(99)00088-8

[26] Yoo D, Lee J, Shin T, Cheon J. Theranostic magnetic nanoparticles. Accounts of Chemical Research. 2011;44(10):863–874. 10.1021/ar200085c 21823593

[27] Reeves D, Weaver J. Magnetic nanoparticle sensing: decoupling the magnetization from the excitation field. Journal of Physics D: Applied Physics. 2014;47(4):045002 10.1088/0022-3727/47/4/045002 24610961 PMC3939079

[28] Atkinson W, Brezovich I, Chakraborty D. Usable frequencies in hyperthermia with thermal seeds. Biomedical Engineering, IEEE Transactions on. 1984;1:70–75. 10.1109/TBME.1984.325372 6724612

[29] Reeves D, Weaver J. Simulations of magnetic nanoparticle Brownian motion. Journal of Applied Physics. 2012;112(12):124311–124311. 10.1063/1.4770322 23319830 PMC3537703

[30] Weizenecker J, Gleich B, Rahmer J, Borgert J. Particle dynamics of mono-domain particles in magnetic particle imaging Magnetic nanoparticles: particle science, imaging technology, and clinical applications Singapore: World Scientific Publishing Co Pte Ltd 2010; p. 3–15.

[31] García-Palacios J, Lázaro F. Langevin-dynamics study of the dynamical properties of small magnetic particles. Physical Review B. 1998;58(22):14937 10.1103/PhysRevB.58.14937

[32] Rogge H, Erbe M, Buzug T, Lüdtke-Buzug K. Simulation of the magnetization dynamics of diluted ferrofluids in medical applications. Biomedical Engineering. 2013;58(6):601–609. 24163220 10.1515/bmt-2013-0034

[33] Martens M, Deissler R, Wu Y, Bauer L, Yao Z, Brown R, et al Modeling the Brownian relaxation of nanoparticle ferrofluids: Comparison with experiment. Medical Physics. 2013;40(2):2303 10.1118/1.4773869 PMC5148099 23387765

[34] Raible M, Engel A. Langevin equation for the rotation of a magnetic particle. Applied Organometallic Chemistry. 2004;18(10):536–541. 10.1002/aoc.757

[35] Yoshida T, Enpuku K. Simulation and quantitative clarification of AC susceptibility of magnetic fluid in nonlinear Brownian relaxation region. Japanese Journal of Applied Physics. 2009;48(12R):127002 10.1143/JJAP.48.127002

[36] Coffey W, Cregg P, Kalmykov Y. On the Theory of Debye and Néel Relaxation of Single Domain Ferromagnetic Particles. Advances in Chemical Physics. 1992;83:263.

[37] Einstein A. Investigations on the Theory of the Brownian Movement. Dover; 1956.

[38] Reeves D, Weaver J. Comparisons of characteristic timescales and approximate models for Brownian magnetic nanoparticle rotations. Journal of Applied Physics. 2015;117(23):233905 10.1063/1.4922858 26130846 PMC4474943

[39] Coffey W, Waldron J, Kalmykov Y. The Langevin Equation. World Scientific; 1996 10.1142/2256

[40] Gardiner C. Handbook of Stochastic Methods. vol. 4 Springer Berlin; 1985.

[41] Usov N, Liubimov B. Dynamics of magnetic nanoparticle in a viscous liquid: Application to magnetic nanoparticle hyperthermia. Journal of Applied Physics. 2012;112(2):023901 10.1063/1.4737126

[42] Kloeden P, Platen E. Numerical Solution of Stochastic Differential Equations. Springer; 1992.

[43] Goya G, Berquo T, Fonseca F, Morales M. Static and dynamic magnetic properties of spherical magnetite nanoparticles. Journal of Applied Physics. 2003;94:3520 10.1063/1.1599959

[44] Chantrell R, Popplewell J, Charles S. Measurements of particle size distribution parameters in ferrofluids. Magnetics, IEEE Transactions on. 1978;14(5):975–977. 10.1109/TMAG.1978.1059918

[45] Khandhar A, Ferguson R, Simon J, Krishnan K. Tailored magnetic nanoparticles for optimizing magnetic fluid hyperthermia. Journal of Biomedical Materials Research. 2012;100(3):728–737. 10.1002/jbm.a.34011 22213652 PMC3266447

[46] Bordelon DE, Cornejo C, Grüttner C, Westphal F, DeWeese TL, Ivkov R. Magnetic nanoparticle heating efficiency reveals magneto-structural differences when characterized with wide ranging and high amplitude alternating magnetic fields. Journal of Applied Physics. 2011;109(12):124904 10.1063/1.3597820

[47] Debye P. Polar molecules. Chemical Catalog Company, Incorporated; 1929.

[48] Reeves D, Weaver J. Comparisons of characteristic timescales and approximate models for Brownian magnetic nanoparticle rotations. Journal of Applied Physics. 2015;117(23):233905 10.1063/1.4922858 26130846 PMC4474943

[49] Shah S, Ferguson R, Krishnan K. Slew-rate dependence of tracer magnetization response in magnetic particle imaging. Journal of Applied Physics. 2014;116(16):163910 10.1063/1.4900605 25422528 PMC4224682

[50] Dieckhoff JH, Yoshida T, Enpuku K, Schilling M, Ludwig F. Homogeneous bioassays based on the manipulation of magnetic nanoparticles by rotating and alternating magnetic fields?a comparison. Magnetics, IEEE Transactions on. 2012;48(11):3792–3795. 10.1109/TMAG.2012.2198797

[51] Yang C, Yang S, Chieh J, Hong H, Hong C, Yang H. Universal Behavior of Biomolecule-Concentration-Dependent Reduction in AC Magnetic Susceptibility of Bioreagents. Magnetics Letters, IEEE. 2012;3:1500104–1500104. 10.1109/LMAG.2012.2183858

